# Association between social support and the severity of positive symptoms in rural community-dwelling patients with schizophrenia during the COVID-19 pandemic

**DOI:** 10.1186/s12888-024-05571-z

**Published:** 2024-02-14

**Authors:** Yudan Liu, Hongying Liu, Siyu Chen, Jidong Ren, Xiaobing Tian

**Affiliations:** 1 School of Public Health, North Sichuan Medical College, No.234 Fujiang Road, 637000 Nanchong, Sichuan China; 2 Nanchong Psychosomatic Hospital Affiliated to North Sichuan Medical College, Nanchong, Sichuan China

**Keywords:** Social support, Positive symptoms, Schizophrenia

## Abstract

**Background:**

This study examined the association between social support and the severity of positive symptoms in rural community-dwelling schizophrenia patients during the COVID-19 pandemic.

**Method:**

The cross-sectional study included 665 rural community-dwelling schizophrenia patients investigated during the COVID-19 pandemic. Social support was measured using the Social Support Rating Scale, and positive symptoms were assessed using the Positive Scale extracted from the Positive and Negative Syndrome Scale. Multiple linear regression was adopted to examine the association of social support with positive symptoms.

**Result:**

The scores for total social support, subjective support, objective support and the use of social support were 28.3 ± 5.9, 16.4 ± 5.2, 6.5 ± 1.4 and 5.4 ± 2.8, respectively. Total social support (*β =* −0.08, 95%CI: −0.13 to −0.02, *P* < 0.01) and subjective social support (*β =* −0.10, 95%CI: −0.16 to −0.04, *P* < 0.01) were significantly and negatively associated with the Positive Scale score after adjustment for confounders. Objective social support (*β =* 0.11, 95%CI: −0.10 to 0.32, *P* = 0.31) and the use of social support (*β =* −0.03, 95%CI: −0.14 to 0.07, *P* = 0.53) were not significantly associated with the Positive Scale score.

**Conclusion:**

The study confirmed the importance of social support, especially subjective support, provided to rural community-dwelling schizophrenia patients during the COVID-19 pandemic. This support should be addressed and strengthened for such patients in emergent events.

## Introduction

The weighted lifetime prevalence of schizophrenia is 0.6% in China [1]. As of 2019, approximately 4.5 million schizophrenia patients were registered in China, with the majority being patients dwelling and managed in communities [2]. Many of these community-dwelling patients lived in rural areas [2]. In China, patients with severe mental disorder, whether in a rural or urban community, receive free medication and community-based mental health services [3]. However, rural areas usually have a poor economic status and inadequate mental health resources, and schizophrenia patients face great stigma [4–6], leading to vulnerable community mental health service systems.

Social support benefits patients’ recovery from schizophrenia, as demonstrated in numerous studies [7, 8], with patients who have greater social support having better medication adherence and lower stigma than other patients [4, 9]. Primary mental health care workers in rural areas have less professional technical ability than those in urban areas, and there are insufficient full-time personnel for ensuring quality patient management [6, 10]. Rural patients fail to collect free medication when they are reluctant to take medicine or are unsupervised or live far from locations designated for drug distribution, leading to poor medication adherence [11]. The stigma of schizophrenia is more severe in rural areas than in urban areas [4] and can affect health-seeking behaviors [12, 13]. A subjective perception of schizophrenia patients, such as a sense of belonging and satisfaction, is important to the patients and can be obtained through community rehabilitative activities [8, 13, 14]. Social support might reduce stigma and create opportunities for patients to gain such subjective perception [4].

During the COVID-19 pandemic, the delivery of and access to mental health services were disrupted by the implementation of social isolation measures and physical quarantining. Schizophrenia patients with cognitive impairment and a lower awareness of risk faced a high risk of pandemic virus infection, and there were two infection clusters in inpatient psychiatric wards in Wuhan, China and South Korea during the early stages of the pandemic, with many patients and medical workers infected [12]. Social panic relating to community schizophrenia patients arose owing to a fear of being infected and the stigma toward people with both quarantine experience and schizophrenia [4, 15]. Schizophrenia patients living in rural communities were thus much vulnerable during the pandemic.

Given the limited medical resource in rural areas as well as the dramatically changed network of social support during COVID-19 pandemic, new evidences are needed to gain a better understanding of social support on rehabilitation to inform an effective intervention scheme. The aim of our study is to identify the association of social support with positive symptoms of schizophrenia among rural community-dwelling patients with schizophrenia in the context of the COVID-19 pandemic and to confirm beneficial factors that promote the effective care of community-dwelling psychosis in an unfavorable environment.

## Methods

### Data and participants

Participants with schizophrenia were selected from the Sichuan Province Comprehensive Management Information Platform for Severe Mental Disorders in 2021. Details of the sampling and data collecting process were made available in a previous cross-sectional study, which was conducted during the COVID-19 pandemic from 1 May to 30 July in 2021 in Yingshan county, Sichuan province, which is located in southwestern China [16]. The data used in the present study were the rural subset of data abstracted from the previous study [16]. Participants met the following inclusion criteria. (1) The participants were diagnosed with schizophrenia according to the International Classification of Diseases (ICD-10) and Chinese Classification of Mental Disorder (CCMD-3). (2) They were at least 1 year post diagnosis. (3) They had been managed in the community for at least half a year and had been prescribed standard medication. (4) They resided in rural areas. Patients who lacked the ability to complete the survey, lacked a clear guardian or had other mental or severe physical disease were excluded. Finally, 665 schizophrenia participants were included in the study. A face-to-face interview was conducted by one physician and four nurses, and details was narrated in previous study [16]. The Institutional Ethics Review Board of the Psychosomatic Hospital Affiliated with the North Sichuan Medical College approved the investigation. All patients and guardians provided informed consent, and the participants participated voluntarily throughout the study.

### Measurements

Social support was measured on the Social Support Rating Scale (SSRS) [17], which comprises 10 items and the three dimensions of objective support, subjective support and the use of social support. The dimension of objective support has three items with which to assess objective, visible and practical support, including direct material assistance, social networks and group relations. The dimension of subjective support includes four items for subjective, experiential or emotional support involving individual emotional experiences and the satisfaction of being respected, supported and understood in society. The dimension of the use of social support comprises three items addressing the support used initiatively by the respondent. A higher score on the SSRS indicates greater support [17]. The SSRS has been applied to populations of patients with schizophrenia and was shown to have good reliability and validity. The Cronbach’s α was 0.6 in this study.

Positive symptoms of schizophrenia were assessed using the Positive Scale extracted from the 30-item Positive and Negative Syndrome Scale (PANSS). The PANSS includes three dimensions, namely a Positive Scale, Negative Scale and General Psychopathology, and each dimension and the total scale have high levels of reliability and validity [18]. The Positive Scale has seven items. Each item is scored from 1 to 7 corresponding to seven increasing levels of psychopathology symptoms, from absence to extreme. A higher score on the scale thus indicates more serious positive psychopathology symptoms. The Cronbach’s α in this study was 0.7.

The participant characteristics investigated in this study were gender, age (< 45 years, 45–59 years, ≥ 60 years), educational levels of the patient and guardian (semi-illiterate, primary school, junior high school or above), marital status (married, single, widowed and other), occupations of the patient and guardian (farmer, job lost or unemployed, other), relationship of the guardian (parent, partner, other), family income level (poor, ordinary, good), participation in rehabilitation activities (no, yes) and medication adherence (no, yes).

### Data analysis

The data were analyzed using SPSS software, version 22.0. Quantitative variables (age and scores on the SSRS and Positive Scale) are presented as means and standard deviations ($\stackrel{-}{x}\pm \text{s}$). Categorical variables (gender, age group, education, marital status, occupation, educational level of the guardian, occupation of the guardian, relationship of the guardian, family income level, rehabilitation activity and medication adherence) are presented as counts and percentage. Differences in the Positive Scale scores between different participants with distinct characteristics were evaluated using a *t*-test or analysis of variance. The correlations between the scores on the SSRS and Positive Scale are presented using Pearson product moment coefficients. The association of social support with positive symptoms was assessed through multiple linear regression. Multivariate regression models were established with model 1, which was not adjusted, and model 2, which was adjusted for the participant characteristics that were significant in the univariate analysis. In all models, the Positive Scale score was taken as the dependent variable and social support as the independent variable, and partial regression coefficients and corresponding 95% confidence intervals (95%CIs) were estimated. In this study, two-tailed *P* values of ≤ 0.05 were considered significant.

## Results

### Sociodemographic characteristics

Table 1 gives the participant characteristics and Positive Scale score between participants with different characteristics. The average age was 51.6 ± 13.9 years. The proportion of female participants was 60.5%. The majority of participants had a low educational level, were married and were farmers or had lost their jobs/were unemployed. Among the participants, 36.1% and 58.3% had poor and ordinary family incomes, respectively. Most of the guardians had a low educational level, were farmers or were unemployed and were a parent or partner. A minority of the participants had participated in community rehabilitation activities and adhered to taking medication.

### Comparison of positive scale score between different participants

The mean Positive Scale score of the 665 participants was 10.1 ± 4.0. There were significant differences between participants according to gender, marital status, family income level, guardian occupation and medication adherence (*P* < 0.05). Female participants and those with single, widowed and other marital status, a poor family income, farmer guardians and nonadherence to medication had significantly higher scores than did other participants.

**Table 1:** Participant characteristics and comparison of Positive Scale score between participants with different characteristics (*N* = 665)

Variables	Participants		Positive Scale	$$ t$$/F	P
	n	Percentage (%)	$\stackrel{-}{x}\pm \text{s}$		
Gender					
Male	263	39.5	9.6 ± 4.0	2.37	0.02
Female	402	60.5	10.4 ± 4.1		
Age					
<45	199	29.9	9.7 ± 3.9	1.80	0.17
45 ~ 59	271	40.8	10.1 ± 3.8		
≥ 60	195	29.3	10.5 ± 4.4		
Educational level					
Semi-illiterate & illiterate	217	32.6	10.5 ± 4.3	2.75	0.06
Primary school	267	40.2	10.1 ± 4.2		
Junior and above	181	27.2	9.6 ± 3.4		
Marital status					
Married	453	68.1	9.8 ± 3.7	2.37	0.02
single, widowed and other	212	31.9	10.6 ± 4.7		
Occupation					
Farmer	244	36.7	9.8 ± 4.0	1.97	0.14
Job lost or unemployed	397	59.7	10.3 ± 4.1		
others	24	3.6	9.1 ± 3.0		
Family income level					
Poor	240	36.1	10.4 ± 4.0	5.97	< 0.01
Ordinary	388	58.3	10.1 ± 4.1		
Good	37	5.6	8.0 ± 2.7		
Guardian educational level					
Semi-illiterate & illiterate	168	25.3	9.7 ± 4.1	0.96	0.38
Primary school	346	52.0	10.2 ± 4.0		
Junior and above	151	22.7	10.2 ± 4.2		
Guardian occupation					
Farmer	403	60.6	10.4 ± 4.3	4.19	0.02
Job lost or unemployed	113	17.0	10.0 ± 3.6		
others	149	22.4	9.3 ± 3.4		
Relation to guardian					
Parent	203	30.5	10.2 ± 4.2	1.06	0.35
Couple	362	54.4	9.9 ± 3.8		
Others	100	15.0	10.5 ± 4.6		
Community rehabilitation activity					
Yes	178	26.8	10.2 ± 3.3	0.21	0.83
No	487	73.2	10.1 ± 4.3		
Medication adherence					
Yes	265	39.8	9.1 ± 2.8	5.48	< 0.01
No	400	60.2	10.8 ± 4.5		
Total			10.1 ± 4.0		

### Correlation between the scores on the SSRS and positive scale

The mean scores for total social support, objective support, subjective support and the use of support were 28.3 ± 5.9, 6.5 ± 1.4, 16.4 ± 5.2 and 5.4 ± 2.8, respectively. The correlations between the scores on the SSRS and Positive Scale are summarized in Table 2. There were significant correlations between the total social support score and the Positive Scale score (*r* = −0.17, *P* < 0.01), and between the subjective support score and the Positive Scale score (*r* = −0.18, *P* < 0.01). There were nonsignificant correlations between the objective support (*r* = −0.001, *P* = 0.97) and use of social support scores (*r* = −0.03, *P* = 0.37) and the Positive Scale score.

**Table 2:** The correlation between the scores on the SSRS and positive scale

Social support	$\stackrel{-}{x}\pm \text{s}$	r	P
Objective	6.5 ± 1.4	-0.001	0.97
Subjective	16.4 ± 5.2	-0.18	< 0.01
Use	5.4 ± 2.8	-0.03	0.37
Total	28.3 ± 5.9	-0.17	< 0.01

### Association between social support scores and positive scale score

Table 3 gives the results of the multiple regression analysis. In the case of unadjusted model 1, the scores for total social support (crude *β* = −0.12, 95%CI: −0.17 to −0.07) and subjective social support (crude *β =* −0.14, 95%CI: −0.20 to −0.08, *P* < 0.01) were significantly and negatively associated with the Positive Scale score. After adjusting for the participant characteristics that were statistically significant in the univariate analysis (gender, marital status, family income level, guardian occupation and medication adherence), the scores for total social support (adjusted *β =* −0.08, 95%CI: −0.13 to −0.02, *P* < 0.01) and subjective support (adjusted *β =* −0.10, 95%CI: −0.16 to −0.04, *P* < 0.01) remained significantly and negatively associated with the Positive Scale score for model 2. The associations of the scores for objective support and the use of social support with the Positive Scale score were nonsignificant for both models 1 and 2.

**Table 3:** Association between social support scores and positive scale score

Social support	Model 1			Model 2	
	β(95%CI)	P		β(95%CI)	P
Objective	-0.004(-0.221, 0.213)	0.97		0.11(-0.10, 0.32)	0.31
Subjective	-0.14(-0.20, -0.08)	< 0.01		-0.10(-0.16, -0.04)	< 0.01
Use	-0.05(-0.16, 0.06)	0.37		-0.03(-0.14, 0.07)	0.53
Total	-0.12(-0.17, -0.07)	< 0.01		-0.08(-0.13, -0.02)	< 0.01

Note: Model 1 was not adjusted for any variable. Model 2 was adjusted for the participant characteristics that were statistically significant in univariate analysis including gender, marital status, family income level, guardian occupation and medication adherence

## Discussion

We investigated 665 rural community-dwelling schizophrenia patients in a county located in southwestern China during the COVID-19 pandemic. In this study, we confirmed that social support was negatively associated with the severity of positive symptoms in this population, and that subjective social support was significantly associated with the Positive Scale score.

### Participants with vulnerable demographic characteristics and poor medication adherence had more severe positive symptoms

In this study, patients who had a low family income level had more severe positive symptoms, and this result was consistent with that of another study conducted among rural schizophrenia patients [19]. Female and unmarried patients had a higher Positive Scale score than did male and married patients in the present study; similar results were also found in previous studies, although without statistical significance [20, 21]. Patients whose guardians were farmers had more severe positive symptoms than did others, similar to a previous indirect finding that caregivers working in the non-agriculture domain had higher social support than others, which was negatively correlated with positive symptoms [22, 23]. In the present study, poor medication adherence was associated with more severe positive symptoms, in agreement with a previous result that antipsychotic-free patients had more prominent positive symptoms, as measured on the Brief Psychiatric Rating Scale, than antipsychotic-treated patients [24]. The results of the present study indicate that community patients with vulnerable demographic characteristics and poor medication adherence had a poor prognosis of positive symptoms and required more attention than others.

### Social support of participants was worse during the pandemic

The patients in the present study had low levels of social support. In a study on cases of remission schizophrenia conducted from May to September 2020, the means total social support, objective support, subjective support and the use of support scores were 30.8, 6.9, 17.2 and 6.7, respectively [25]. The scores for the total scale and the three dimensions of social support in this study and a previous study conducted during the pandemic were lower than those in studies of outpatient and inpatient schizophrenia cases conducted outside the pandemic period [26, 27], which indicated that social support of those patients was worse during the pandemic.

### Social support was beneficial to participants’ recovery

The present study confirmed the association of social support with favorable positive symptoms in schizophrenia patients and was thus consistent with an earlier study [23]. In addition, the results were similar to the results of other studies in which perceived social support from the family domain was higher in the remission group of schizophrenia patients than in the no-remission group and was independently associated with symptom remission [7], and that schizophrenia patients out of remission had more unmet needs than those with a stable remission status [28]. Among the three dimensions of support, subjective support was significantly and inversely associated with the participants’ positive symptoms in the present study, similar to the results of a previous study [23]. A potential explanation is that subjective support, such as caring and understanding, indirectly promotes individuals’ physical and mental health through social psychological mechanisms such as enhancing a subjective sense of mastering to others and belonging [29]. For instance, subjective perceptions such as understanding, caring and belonging in schizophrenia are factors that contribute to recovery [30], and being respected and valued by others is a predictor of subjective recovery in psychosis [31].

### Rural participants may have more barriers to accessing social support during the pandemic

However, patients living in rural China may have encountered more barriers to accessing social support during the pandemic than those living in urban areas. Patients feel that they are being helped and report feeling satisfied when they benefit from medical insurance, a disability certificate that reduces costs and a basic living allowance [32], yet rural patients receive inadequate financial subsidies [33]. Stigma can hinder a schizophrenia patient from making contact with others [32], and schizophrenia patients with internalized stigma have lower relational satisfaction, lower relational esteem and higher fears of relationships or relational anxiety than schizophrenia patients without internalized stigma [34]; additionally, stigma is more common among patients living in rural areas than among those in urban areas [4]. Furthermore, patients gain friendships and the subjective support of a sense of belonging, respect and satisfaction through community rehabilitative activities, yet such activities might stop during a pandemic [13]. During the COVID-19 pandemic, remote options such as wearable devices or online services have been adopted as alternative measures to monitor vital signs and treat common mental-health disorders [15, 35]. Such remote options may be more difficult to implement and have lower acceptance in rural areas than in urban areas, given the worse economy, lower educational level and customs of seeking medical advice in rural areas. A survey conducted in rural China during the pandemic revealed that the proportion of telemedicine use was low owing to the barriers of a lack of equipment and knowledge regarding telemedicine access and a lack of trust in and demand for telemedicine [36]. In addition, health literacy is positively correlated to COVID-19 knowledge, and residents in rural areas have lower health literacy but a lower level of health education on COVID-19 than residents in urban areas [37, 38].

### Summary

In summary, the subjective social support provided to rural schizophrenia patients was poor during the pandemic and not conducive to the relief of positive symptoms. Severe positive symptoms might aggravate stigma toward schizophrenia patients and affect social support [4, 23, 39]. Moreover, it has been shown that emotional support is more beneficial to overall health and psychological well-being among low-income people than among high-income people [40]. A subjectively supportive environment is thus crucial to rural community-dwelling schizophrenia patients and should be strengthened in emergencies. Objective support was not associated with positive symptoms in the present study, similar to a previous study that found no correlation of instrumental social support with positive symptoms [23]. Although a previous study found that the use of support, such as a willingness to ask for help, is correlated with positive symptoms [31], subjective support alone was associated with positive symptoms in this study.

### Limitations

Our study has several limitations. The aim of our study was to analyze the causal relationship from social support to positive symptoms. However, due to the design, cross-sectional study and inversion of causality might occur, that was, schizophrenia patients who had milder positive symptoms might experience less stigma and subjectively be more satisfied with their social support. Thus, prospective studies are needed to verify the causality in the future. In addition, the social support provided to participants before the COVID-19 pandemic was not investigated and thus unknown, such that we could not compare social support before and during the pandemic. However, the present study highlights the effect of social support, particularly subjective support, on community-dwelling schizophrenia patients in a rural and pandemic environment.

## Conclusion

The present study confirmed that social support, especially subjective support, was negatively associated with the severity of positive symptoms among rural community-dwelling schizophrenia patients during the COVID-19 pandemic. This result highlights the importance of providing social support, especially subjective support, to rural community-dwelling schizophrenia patients to ensure sustainable community-based mental health service.

## Data Availability

The datasets used during the current study are available from the corresponding author on reasonable request.
